# Hepatitis C Pretreatment Profile and Gender Differences: Cognition and Disease Severity Effects

**DOI:** 10.3389/fpsyg.2019.02317

**Published:** 2019-10-15

**Authors:** David Pires Barreira, Rui Tato Marinho, Manuel Bicho, Isabel Flores, Renata Fialho, Sílvia Ouakinin

**Affiliations:** ^1^Clínica Universitária de Psiquiatria e Psicologia Médica, Faculdade de Medicina, Universidade de Lisboa, Serviço de Gastrenterologia e Hepatologia, Centro Hospitalar Lisboa Norte-Hospital de Santa Maria, Lisbon, Portugal; ^2^Faculdade de Medicina, Universidade de Lisboa, Serviço de Gastrenterologia e Hepatologia, Centro Hospitalar Lisboa Norte-Hospital de Santa Maria, Lisbon, Portugal; ^3^Laboratório de Genética, Faculdade de Medicina, Instituto de Saúde Ambiental, Universidade de Lisboa, Lisbon, Portugal; ^4^ISCTE, IUL, Centro de Investigação em Estudos Sociais, Lisbon, Portugal; ^5^Immunopsychiatric Clinic, Research and Development, Sussex Partnership NHS Foundation Trust, Brighton and Hove, United Kingdom; ^6^Clínica Universitária de Psiquiatria e Psicologia Médica, Faculdade de Medicina, Universidade de Lisboa, Lisbon, Portugal

**Keywords:** hepatitis C, neurocognition, anxiety, depression, gender, disease severity

## Abstract

**Background:**

The hepatitis C virus (HCV) is known to infect the brain, however, the findings based on associated neuropsychiatric syndrome are controversial and the association itself remains unclear. Gender research in HCV infection is limited, failing to integrate the role of gender differences in neurocognitive syndrome. The aim of this study was to characterize psychological and neurocognitive profiles in HCV-infected patients before treatment and to explore gender differences in those profiles, as well as the impact of disease severity.

**Methods:**

A total of 86 patients diagnosed with chronic hepatitis C were included. Depression and anxiety were assessed using Hamilton anxiety scale (HAM-A), Hamilton depression scale (HAM-D), Beck Depression Inventory (BDI). For cognition, a neuropsychological battery to measure attention, concentration and memory was used, and executive function components validated for the Portuguese population was also used before starting treatment. To identify the disease severity, platelet ratio index, and FibroScan^®^ were used.

**Results:**

A statistically significant gender effect was found on HAM-A (*B* = 0.64, CI: 0.17–1.11) and HAM-D (*B* = 0.62, CI: 0.14–1.09), with women scoring higher compared to men. Regarding neuropsychological scores, significant differences between gender were identified in executive functions measured by Trail Making Test (TMT B) (*B* = 0.48, CI: 0.02–0.97), TMT B-A (*B* = 0.26, CI: −39.2 to −3.7) and in digit span total (*B* = −0.52, CI: −1.0 to −0.04), with women performing worse than men. Controlling for years of substance dependence, TMT-B and TMT B-A showed significant gender differences. Regarding the presence or absence of substance dependence, only HAM-A and HAM-D remained significant. For categorical variables, Digit Span Total was also influenced by gender, with women being more likely to be impaired: odds ratio (OR) = 7.07, CI: 2.04–24.45), and a trend was observed for Digit Span Backward (OR = 3.57, CI: 1.31–9.75). No significant differences were found between disease severity and neurocognitive performance.

**Conclusion:**

Data suggest that gender has an influence on depression, anxiety and cognitive functions with women showing greater impairment compared with men. This effect seems to be influenced by substance dependence.

## Introduction

Hepatitis C virus (HCV) is one of the most important infections worldwide, with 71 million people infected (Polaris Observatory et al., 2017). Despite the advent of HCV therapies and high rates of cure, if not treated, HCV acute infection tends to progress to chronic state, hepatic cirrhosis (10 to 40%) and hepatocellular carcinoma, which tend to be clinical indications for liver transplant (1 to 5% per year) (Monaco et al., 2015).

Hepatitis C virus patients frequently present neuropsychiatric symptoms, including fatigue, anxiety, depression, and neurocognitive impairment, that have been associated with negative treatment outcomes (Monaco et al., 2015; Dirks et al., 2017; Yarlott et al., 2017). Several studies reported that HCV associated neuropsychological impairments are independent of liver disease stage (excluding cirrhosis) (Monaco et al., 2015; Yarlott et al., 2017), suggesting that, in chronic patients, HCV infection itself might directly cause cerebral dysfunction, contributing to cognitive deficits in patients (Grover et al., 2012). Therefore, the neurocognitive profile of these patients before treatment needs to be addressed, due to the possible implications on the treatment course (e.g., non-adherence-like behavior due to cognitive dysfunction). Additionally, it is important to address other risk factors that can have a negative impact on cognition. Despite the eventual effects of antiviral medication on cognition, the high rate of substance abuse and the prevalence of psychiatric disorders among HCV-infected patients are important factors associated with cognitive impairment that compromises adherence and treatment efficacy (Więdłocha et al., 2017).

Most of the patients with HCV consistently report a significant reduction in health-related quality of life, in comparison with healthy controls (Adinolfi et al., 2017). The psychiatric symptomatology in chronic hepatitis C, in particular depression, is twice as high as in the general population, and it is probably one of the major factors for decreasing quality of life, treatment discontinuation and/or negative treatment outcomes (Monaco et al., 2015).

According to several studies, it has been hypothesized that depression and cognitive impairment might be associated with HCV neurotoxicity (Adinolfi et al., 2015). Recent studies with interferon-free treatment showed that HCV patients presented neuropsychiatric symptoms like fatigue, insomnia, anxiety, depression, and cognitive dysfunction (Gritsenko and Hughes, 2015; Smith et al., 2015; Chasser et al., 2017), supporting the HCV neurotoxicity hypothesis.

In untreated HCV patients, emotional distress and depressive disorders suggest the role of HCV itself in the onset of these neuropsychiatric manifestations (Alavi et al., 2012). In a recent review, the “hepatitis C brain syndrome” was identified as being composed of cognitive impairment, fatigue, and depression. This syndrome is probably generated by peripheral immune responses, affecting the central nervous system (CNS) by a neuroinflammatory response associated with CNS HCV infection and also by negative life events and other psychogenic stressors (Yarlott et al., 2017).

Neurocognitive impairment, one of the most common extrahepatic manifestations of HCV, can lead to subtle changes in processing speed, memory, and cognitive performance (attention, concentration, psychomotor speed, and verbal fluency), and up to 50% of HCV-infected patients can develop clinical or subclinical manifestations of this dysfunction (Lowry et al., 2010; Ferri et al., 2016). Cognitive impairments were previously thought to be associated with the development of hepatic encephalopathy (Gaeta et al., 2013). However, their presence was demonstrated in the absence of advanced liver disease, as well as in the absence of human immunodeficiency virus (HIV) coinfection, depression or substance dependence (Weissenborn et al., 2004; Kuhn et al., 2017; Yarlott et al., 2017). HCV is known to infect the brain; however, findings from studies on associated neurocognitive changes are controversial and it remains unclear whether HCV eradication improves neurocognitive performance (Kuhn et al., 2017).

Regarding gender differences, women with HCV seem to experience stigma, concerns about confidentiality, treatment side effects and lack of engagement with health services, more so than men (Harris and Rhodes, 2013). However, the gender differences research in symptoms profile, as well as in treatment response and treatment adherence, is scarce, albeit crucial to improving medical and psychological integrated interventions.

This study aimed to characterize psychological and neurocognitive profiles in HCV-infected patients before treatment, and to describe gender differences as well as the impact of disease severity in those profiles.

## Materials and Methods

This research is part of a larger longitudinal protocol including two assessment times, before and after HCV antiviral medication. It was submitted to and approved by the Centro Hospitalar Universitário Lisboa Norte Ethics Committee (Ref. 0536) and the Portuguese Data Protection Authority (No. 6331/2012).

If eligible, subjects were informed about the nature of the study and their voluntary cooperation. After confidentiality assurance, written informed consent was obtained.

### Recruitment of Participants

A total of 86 patients diagnosed with chronic hepatitis C were selected from an outpatient clinic through a convenience procedure (Viral Hepatitis Clinic at the Hospital de Santa Maria, Lisbon).

The inclusion criteria were: minimum formal schooling completed with success (at least 4 years education or fluency in reading and writing); ages between 18 and 65 years-old; diagnosis of chronic hepatitis C with detectable HCV RNA viral load for at least 6 months. The exclusion criteria were: presence of neurological or psychiatric disorders that may induce cognitive deficits; major depression according to DSM-V; consumption of opiates, cocaine and/or other recreational drugs in the 6 months prior to the beginning of the assessment; use of medication that may interfere with the study objectives; history of neurological, infectious or tumoural pathology or systemic illness with an impact at CNS level; a Mini-Mental State Examination (MMSE) score below the cut-off for dementia in the Portuguese population; hepatic cirrhosis or severe physical deterioration incompatible with the psychologic and neuropsychologic assessments.

### Assessment and Evaluation

A semi-structured interview was performed to collect sociodemographic and clinical data.

### Psychiatric Evaluation

#### Depression

Depressive symptoms were assessed using a patient-rated questionnaire and a clinician administrated one, to control for the bias of self-reporting.

#### Hamilton Depression Scale (HAM-D)

Depression was assessed using the 17-item HAM-D (Hamilton, 1960). The Hamilton scale was developed for patients diagnosed with depression, to measure its intensity and to identify depressive symptoms profiles. We used the cut-off scores validated for the Portuguese population (Vaz Serra, 1972).

#### Beck Depression Inventory (BDI)

Beck published the “Inventory for Measuring Depression” in 1961, representing the first self-assessment questionnaire for the study of depression (Beck et al., 1961). The inventory comprises 21 symptoms that cover multiple aspects of depressive manifestations, not only affective but also cognitive, motivational and behavioral. In this study, the translated and validated Portuguese version was used (Vaz Serra, 1972).

### Anxiety

#### Hamilton Anxiety Scale (HAM-A)

Anxiety was assessed using the 14-item HAM-A (Hamilton, 1959). The Hamilton scale for anxiety comprises 14 symptoms covering the most characteristic manifestations of the anxious syndrome, both in terms of “psychic” (items 1 to 6) and “somatic” (items 6 to 14) symptoms. This scale is an instrument that has long been validated by several studies and measured for different Portuguese populations (Vaz Serra, 1972).

### Neuropsychological Evaluation

#### Mini-Mental State Examination (MMSE)

The MMSE is one of the most widely used brief instruments for the clinical evaluation of global cognitive state in adults (Folstein et al., 1975). The MMSE is used for the evaluation of the mental state and screening of dementia. It is a 30-point questionnaire, measuring the following areas of cognitive function: time orientation, spatial orientation, registration, attention and calculation, recall, language, repetition, and visual construction. The normative cut-off values for the Portuguese population adjusted to education were used (Guerreiro et al., 1994).

#### Trails Making Test (TMT)

The TMT is an instrument that measures executive functions (Reitan, 1958). It consists of two parts. In Part A, the subject is instructed to connect a set of 25 numbers as fast as possible, while still maintaining accuracy. In Part B, the subject is instructed to connect numbers sequentially with letters. Scoring is expressed in terms of the time (in seconds). The TMT B-A score (calculated as the difference between TMT-B and TMT-A, to remove the individual motor speed element from the task and the composite) is considered a measure of cognitive flexibility relatively independent of manual dexterity (Misdraji and Gass, 2010). The Portuguese-validated version was used (Cavaco et al., 2013).

#### The Battery of Lisbon for the Assessment of Dementia (BLAD)

The BLAD is a comprehensive neuropsychological battery evaluating multiple cognitive domains and validated for the Portuguese population (Guerreiro, 1998). Tests of interest for the present study were Digit Span (DS) and Logical Memory (immediate and delayed recall). DS is a test that evaluates immediate memory (Digit Span Forward), working memory (Digit Span Backward), and attention and concentration (Digit Span Total). Logical Memory (immediate or delayed recall) evaluates verbal memory and learning. The participants below education and age-adjusted values for the Portuguese population (1 standard deviation) were considered impaired. A cut-off value of 1 standard deviation was adopted, considering that the use of the cut-off value of 1.5 standard deviations (Petersen et al., 1999) could exclude subjects that, from a clinical point of view, suffered from mild cognitive impairment (Winblad et al., 2004; Coelho et al., 2017).

### Disease Severity

#### Aspartate Aminotransferase (AST) to Platelet Ratio Index (APRI)

This APRI is among the best-validated methods for predicting HCV progression (Wai et al., 2003). The lower the APRI score (<0.5), the greater is its negative predictive value (and ability to rule out cirrhosis); the higher the value (>1.5), the greater the positive predictive value (and ability to rule in cirrhosis). The APRI helps with diagnosis based on the platelet count and AST level. The APRI alone is likely not sufficiently sensitive to rule out the significant disease. Some evidence suggests that the use of multiple indices in combination, such as APRI plus findings on transient hepatic elastography, or an algorithmic approach, may result in higher diagnostic accuracy than using APRI alone (Chou and Wasson, 2013).

#### Transient Hepatic Elastography (TE)

Liver stiffness measurement by TE was performed according to published recommendations, using the M probe of FibroScan^®^. The results were expressed in kPa. Only procedures with at least 10 validated measurements, >60% success rate and an interquartile range inferior to 25% of the median value were considered reliable (Pons et al., 2017). The standard 12.5 kPa for the diagnosis of cirrhosis was used (Manuel Echevarría et al., 2015).

#### Statistical Analysis

Results were analyzed using descriptive statistics, including percentages to describe categorical variables and means and standard deviations for continuous ones. Spearman correlation were used to analyze the eventual relation between HCV infection duration and cognitive/psychological evaluation results.

Demographic, clinical, psychological and neuropsychological data were analyzed using independent *t*-tests or linear regression models for dependent continuous variables, as normality was achieved in every distribution. Data were tested for normality using the Shapiro–Wilk test (Ghasemi and Zahediasl, 2012). The continuous variables were all standardized to the mean and standard deviation (*Z* score), and the reported B values represent the standardized coefficients or the dimension of the effect. Every B value between 0.2 and 0.5 was considered to be presenting a relevant effect, and above 0.5 were considered to be of medium dimension. Although the significance (*P*-value) is presented and the significance value was established at a two-tailed threshold of <0.05, all interpretation is based on the concomitant values of the coefficient B and the *P*-value, following recent discussion on the interpretation bias caused by the sole interpretation of the latter (Sullivan and Feinn, 2012; Lakens et al., 2018).

For categorical analysis, chi–squared tests (with the report of Cochran’s and Mantel-Haenszel statistics (odds ratio) were used when just one independent variable was being tested or logistic regression models, when there was more than one independent variable. The values reported for this analysis are the ORs, with a reference neutral value of 1. Once again, a balanced analysis between the odds ratio and the respective *P*-value was reported. Odds ratio superior to 1.5 or inferior to 0.75 were considered as relevant. All the neuropsychological tests were categorized with the value of 1 for defect and 0 for the absence of defect, and women were categorized as 1 and men as 0.

The variables related to disease severity (APRI and FibroScan^®^ values) are both continuous. Patients with a level of APRI and FibroScan^®^ value superior to 2.5 standard deviations of the respective mean were excluded from the sample, as their level of disease severity was found to be compromising the normality of the analysis (Leys et al., 2013). Three males fell under this condition.

Results are presented controlling for years of substance dependence (A.Beta1) and the presence or absence of previous illicit drugs consumption (A.Beta2), as a statistically significant difference between males and females was found. Beta (B) scores represent the influence of the gender over the psychological or neuropsychological measures and the respective standard deviations for a significance at 95%. Beta values higher than 0.20 represent an effect at the sample level.

The reported values are ORs, adjusted ORs for the number of years of substance dependence (A.ORs) and the presence or absence of illicit drugs consumption (A.OR1); a confidence interval of 95% was established.

Statistical analyses were performed using IBM SPSS Statistics 23 for Windows (2016; IBM Corp, Armonk, NY, United States). The statistical methods of this study were reviewed by Isabel Flores, senior consultant in biostatistics.

## Results

### Demographic and Clinic Characterization Data

The sample consisted of 86 HCV-infected patients, 63 men, mean age 47.7 ± 9.9 and 23 women (48.0 ± 8.8), submitted to psychological and neuropsychological assessments. There were no statistically significant differences between genders related to age, diagnosis time, civil status, education, employment, psychiatric antecedents, years of substance dependence, psychoactive substances, alcohol use or smoking. There were statistical differences in substance dependence (89.5% for men and 50% for women, *P* < 0.001) (Table 1).

**TABLE 1:** Demographic and personal characterization with gender comparison.

	**Females, *n* = 23**	**Males, *n* = 63**	***P***
Age	48.0 ± 8.8	47.7 ± 9.9	0.87
Time diagnosis, in yr	9.9 ± 8.6	7.5 ± 7.3	0.24
Education, in yr	9.9 ± 3.3	9.6 ± 4	0.78
**Civil status**			0.9
Single	18.2%	18.3%	
Married	50%	53.3%	
Divorced/separated	27.3%	26.7%	
Widowed	4.5%	1.7%	
**Employment**			0.83
Employed	54.5%	53.3%	
Unemployed	31.8%	38.3%	
Medical leave	4.5%	1.7%	
Retired	9.1%	6.7%	
Psychiatric antecedents	27.3%	11.7%	0.08
Substance dependence	50%	89.5%	0.001^a^
**Psychoactive substances**			0.40
Heroin	36.4%	43.4%	
Cocaine	9.1%	22.6%	
Heroin + Cocaine	18.2%	5.7%	
Marijuana	36.4%	28.3%	
Years of substance dependence	17.0 ± 9.1	17.2 ± 10.7	0.97
**Alcohol**			0.41
No	86.4%	78.3%	
<10 g/day	13.6%	20%	
≥10 g/day	4.5%	3.3%	
**Smoking**			0.55
No	45.5%	33.3%	
<10 cigarettes/day	31.8%	43.3%	
≥10 cigarettes/day	22.7%	23.3%	

*^*a*^*P* < 0.01.*

### Differences in Psychological and Neuropsychological Tests by Gender

There were two levels of analyses: the effect of gender on the continuous variables, and the effect of gender on each neuropsychological indicator classified in two categories - with or without cognitive defect (Tables 2, 3). A linear regression model was used to evaluate the gender effect on psychological and neuropsychological performance.

**TABLE 2:** Gender effect in psychological and neuropsychological evaluations.

	**Females, *n* = 23**	**Males, *n* = 63**	**Beta (95% C.I.)**	**A.Beta1 (95% C.I.)**	**A.Beta2 (95% C.I.)**
HAM-A	14.3 ± 5.2	10.5 ± 6.0	0.64(0.17to1.11)b	0.81(0.35to1.29)b	0.72(0.22to1.24)b
HAM-D	10.2 ± 4.2	7.3 ± 4.6	0.62(0.14to1.09)a	0.74(0.26to1.24)b	0.65(0.13to1.16)a
BDI	8.7 ± 8.1	7.8 ± 8.4	0.11(−0.38to0.60)	0.12(−0.39to0.63)	0.23(−3.0to0.77)
TMT-A	50.3 ± 6.6	44.6 ± 13.6	0.39(−0.36to0.15)	0.36(−0.16to0.88)	0.24(−0.28to0.77)
TMT-B	134.6 ± 49.8	111.1 ± 49.8	0.48(0.02to0.97)a	0.53(0.02to1.05)a	0.31(−0.20to0.83)
TMT B-A	84.3 ± 35.2	62.8 ± 35.9	0.26(-39.2to-3.7)a	0.2(-39.9to-2.6)a	0.17(−33.2to4.28)
Digit span forward	5.3 ± 1.4	5.9 ± 1.2	−0.44(−0.92to0.04)	−0.45(−0.96to0.05)	−0.21(−0.72to0.30)
Digit span backward	3.2 ± 0.9	3.7 ± 1.1	−0.44(−0.92to0.04)	−0.28(−0.78to0.21)	−0.25(−0.77to0.27)
Digit span total	8.6 ± 2.0	9.7 ± 2.1	-0.52(-1.0to-0.04)a	−0.44(−0.94to0.06)	−0.27(−0.77to0.23)
Logical memory (immediate recall)	7.5 ± 2.6	6.8 ± 2.6	0.24(−0.25to0.73)	0.33(−0.18to0.85)	0.31(−0.22to0.84)
Logical memory (delayed recall)	6.6 ± 2.1	5.9 ± 2.4	0.03(−0.52to0.46)	0.05(−0.20to0.25)	−0.08(−0.63to0.45)

*Data is presented as mean ± standard deviation, unless otherwise indicated. ^*a*^*P* < 0.005; ^*b*^*P* < 0.001. BDI, beck depression inventory; C.I., confidence interval; HAM-A, hamilton anxiety scale; HAM-D, hamilton depression scale; TMT, trails making test.*

**TABLE 3:** Association between gender and neuropsychological results, categorized as with or without defect.

		**Males (0), *n* = 63**	**Females (1), *n* = 23**	**ORs (95% C.I.)**	**A.ORs (95% C.I.)**	**A.OR1 (95% C.I.)**
TMT-A	No defect (0)	83.3%	83%	1.05	0.96	1.07
	Defect (1)	16.7%	17%	(0.94–3.76)	(0.25–3.63)	(0.26–4.27)
TMT-B	No defect (0)	73.3%	52.2%	2.5	2.48	1.98
	Defect (1)	26.7%	47.8%	(0.92–6.84)	(0.87–7.07)	(0.67–5.88)
Digit span forward	No defect (0)	88.3%	73.9%	2.7	2.35	2.05
	Defect (1)	11.7%	26.1%	(0.78–9.04)	(0.65–8.44)	(0.54–7.86)
Digit span backward	No defect (0)	73.3%	43.5%	3.57^a^	2.49	2.82
	Defect (1)	26.7%	56.5%	(1.31–9.75)	(0.85–7.27)	(9.62–8.31)
Digit span total	No defect (0)	91.7%	60.9%	7.07^b^	6.86^b^	5.77^b^
	Defect (1)	9.3%	29.1%	(2.04–24.45)	(1.88–24.96)	(1.53–21.88)
Logical memory (immediate recall)	No defect (0)	26.7%	47.8%	0.39	0.28^a^	0.39
	Defect (1)	73.3%	51.2%	(0.14–1.07)	(0.09–0.83)	(0.13–1.16)
Logical memory (delayed recall)	No defect (0)	35%	39%	0.84	0.720	0.66
	Defect (1)	65%	61%	(0.31–2.25)	(0.95–1.02)	(0.22–1.20)

*^*a*^*P* < 0.005; ^*b*^*P* < 0.001. OR, odds ratio; TMT, trails making test.*

Regarding gender, it was possible to identify significant effects on HAM-A (*B* = 0.64, confidence interval (95% C.I.): 0.17–1.11), with women having higher scores on anxiety. The same type of effect was found on HAM-D, with women obtaining a higher significance (*B* = 0.62, 95% C.I.: 0.14–1.09) in depression. No difference between genders was found on the BDI.

In neuropsychological tests, we found significant differences between gender in executive functions measured by TMT-B (*B* = 0.48, 95% C.I.: 0.02–0.97) and in TMT B-A (*B* = 0.26, 95% C.I.: −39.2 to −3.7), with women performing worse.

Digit Span Total was also influenced by gender (*B* = −0.52, 95% C.I.: −1.0 to −0.04), with women having a lower score. Controlling for years of substance dependence, Digit Span Total was no longer significant; however, TMT-B and TMT B-A continued showing gender differences. Controlling for the presence or absence of substance abuse, only HAM-A and HAM-D remained significant.

The duration of the infection did not influence psychological or cognitive results. In fact, we performed a correlation analysis (spearman) between time since the diagnosis and neurocognitive/psychological variables and we only found a weak correlation with TMT B-A (*r* = −0.252 *p* = 0.027).

To evaluate the association between gender and neuropsychological results, categorized as with or without defect analysis, logistic regression models were used.

In terms of the odds ratio, Digit Span Total shows a very relevant result, with women having a 7-times higher possibility of scoring at an abnormal level in this indicator than men (ORs = 7.07, 95% C.I.: 2.04–24.45). After adjustment, the ORs remained significant and with a relevant dimension (A.ORs = 6.86, 95% C.I.: 1.88–24.96; A.OR1 = 5.77, 95% C.I.: 1.53–21.88). For Digit Span Backward, a gender effect was significant only in ORs (ORs = 3.57, 95% C.I.: 1.31–9.75), but was lost after adjustments.

Another relevant difference was found in Logical Memory (immediate recall) OR adjusted for years of substance dependence, where women showed a higher percentage of normal results, presenting a lower possibility of verbal memory and learning defect (A.ORs = 0.28, 95% C.I.: 0.09–0.83). All the other indicators showed no relevant differences between genders.

Although not reaching a significant level, ORs in TMT-B and Digit Span Forward showed a trend to a worse performance in women, with a probability up to 2.5-times higher for a defect, facing men.

### Disease Severity, Gender, Psychological, and Neuropsychological Measures

The possibility of a defect was assessed using as predictors gender, APRI, and FibroScan^®^ values, as measures of disease severity. For psychological dimensions, results were obtained following a multivariable linear regression model and the values reported are the standardized coefficients of the variables (Beta); the Nagelkerke (*R*^2^) represents a measure of goodness of fit. For neuropsychological ones, the indicators were codified as “with defect” (1) and “without defect” (0), and a multiple logistic binary regression was performed (Table 4). The mean APRI score for our study population was 0.77 ± 0.68 and for fibroscan was 8.35 ± 4.32. In APRI, as a predictor of disease progression, individuals were below the value representative of cirrhosis (<1.5), as well as in fibroscan evaluation (<12.5 Kpa).

**TABLE 4:** Association between gender, psychological and categorized neuropsychological results, controlling for disease severity.

**Psychological**	**Gender** **Beta**	**APRI** **Beta**	**FibroScan^®^** **Beta**	***R*^2^**
	**(95%C.I.)**	**(95%C.I.)**	**(95%C.I.)**	
HAM-A	0.62^a^	−0.33	0.05	0.13
	(0.12–1.09)	(−6.79–0.13)	(−0.17–3.44)	
HAM-D	0.56^a^	−0.23	0.07	0.12
	(0.09–1.06)	(−0.64–0.51)	(−0.23–2.8)	
BDI	0.21	−0.25	0.01	0.04
	(−0.26–6.94)	(−0.59–0.09)	(−0.27–2.35)	

**Neuropsychological**	**Gender**	**APRI**	**FibroScan^®^**	**Nagelkerke**
	**OR**	**OR**	**OR**	**(*R*^2^)**

TMT-A	0.83	0.39	1.20	0.05
	(0.19–3.59)	(0.09–1.56)	(0.56–2.58)	
TMT-B	3.04	0.68	0.51	0.15
	(0.99–9.38)	(0.27–1.72)	(0.24–1.72)	
Digit span forward	3.62	0.86	1.46	0.96
	(0.95–13.75)	(0.27–2.72)	(0.58–2.51)	
Digit span backward	3.60^a^	1.0	1.1	0.10
	(1.24–10.49)	(0.44–2.23)	(0.57–1.84)	
Digit span total	8.01^b^	0.81	0.95	0.23
	(2.01–31.97)	(2.36–2.78)	(0.24–1.52)	
Logical memory	0.39	0.82	1.04	0.05
(immediate recall)				
	(0.13–1.13)	(0.37–1.80)	(0.58–1.87)	
Logical memory	0.91	1.48	0.90	0.02
(delayed recall)				
	(0.31–2.63)	(0.65–3.37)	(0.51–1.59)	

*^*a*^*P* < 0.005; ^*b*^*P* < 0.001. C.I., confidence interval.*

Results showed that disease severity has no relevant effect on any score and only gender had a small predictive capacity for HAM-A (*B* = 0.62, 95% C.I.: 0.12–1.09; *R*^2^ = 0.13) and for HAM-D (*B* = 0.56, 95% C.I.: 0.09–1.06; *R*^2^ = 0.12), confirming what had already been reported from the previous analysis.

Digit Span Total provided the most relevant result (Nagelkerke *R*^2^ = 0.23) fully explained by gender, and once again disease severity seemed not to have influence on this score. The OR of 8.01 showed that women have a possibility of about 8 times higher for being classified as with defect on this indicator (*B* = 8.01, 95% C.I.: 2.01–31.97; *R*^2^ = 0.23). In the presence of disease severity, the Digit Span Backward had a 3.6 higher chance of being classified as a defect (*B* = 3.60, 95% C.I.: 1.24–10.49; *R*^2^ = 0.10), in the same line of what has been described before.

## Discussion

Gender research in HCV infection is limited, failing to integrate the role of gender in neurocognitive syndrome. To the best of our knowledge, our study is the first to explore the gender effect in pretreatment HCV-infected patients.

According to our results using clinician-rated instruments (HAM-A and HAM-D), women presented mild levels of anxiety and depression and men showed only mild levels of anxiety. In the self-evaluation questionnaire (BDI), men and women scored below the cut-off level for the diagnosis of clinical depression, ultimately due to a devaluation in emotional self-report.

On neuropsychological performance in pretreatment HCV-infected patients, the gender effect was even more evident. Domains, such as attention, concentration, and memory (short-term and working memory), the ability to shift strategy, executive functions and visual-spatial working memory, showed significant differences with women being more impaired than men. Additionally, when participants were categorized as with or without defect, women performed poorly in short-term and in working memory and attention. This gender effect for executive functions was lost when controlling for substance dependence but was maintained and stood out as being significant for attention, concentration and working memory.

These results can be explained due to the gender impact of the disease. Previous findings suggest a significantly higher impact on the quality of life and also a higher burden of the hepatitis C diagnosis in women (Modabbernia et al., 2013). In fact, in the general population, the prevalence of depressive disorders is higher in women (World Health Organization [WHO], 2016), with a greater burden of mood and anxiety disorders and a lifetime prevalence around 21% compared to 13% in men (Kessler et al., 1993).

The impact of psychosocial factors associated with these disorders are also higher in women, and the risk of developing mood disorders associated to adverse life events imply several contributing factors, such as family, work, environmental-related stressors, poor social support or childhood abuse (Alexander, 2007). Biological sex-related variables are also a major determinant of risk for depression, as well as coping with a chronic disease, such as hepatitis C (Kessler et al., 1993). These factors might, perhaps, enhance vulnerability to the neurotoxic effects of the virus in addition to the burden of the disease (Dannehl et al., 2014). In subsequent analysis, our data replicated previous findings of gender effect, with women presenting higher scores in depression and anxiety, even when controlling for drug use and years of substance dependence. Years of drugs misuse seems to be important for verbal memory and learning, with men scoring worse than women. Although these results highlighted the impact of several years of drug use, they do not fully explain the impairment in cognitive functions in our sample. Therefore, these results are in agreement with several literature reports that have highlighted the CNS toxicity of HCV (Forton et al., 2001).

Disease severity does not seem to influence gender effect, or the profile displayed by patients, perhaps due to its low variability in our sample – Fibroscan (men 8.42 ± 4.22; women 8.42 ± 3.98); APRI (men 0.79 ± 0.69; women 0.55 ± 0.31).

The psychological and neurocognitive characterizations of these patients before treatment assumes particular significance. The importance of preventing and treating psychological comorbidities, such as depression and anxiety, can lead to a better quality of life and better overall functioning during treatment. Impairments due to psychological or neurocognitive symptoms are likely to interfere with the way patients experience and cope with treatment (Barreira et al., 2019). The use of a personalized and patient-centered approach, promoting a deeper understanding of disease state and needs, is also desirable (Schaefer et al., 2012; Modabbernia et al., 2013).

Moreover, factors like gender, depression, psychiatric diagnosis in general, illicit drug use, HIV coinfection, treatment regimen, and hemoglobin levels are variables identified as being significantly and consistently associated with adherence/non-adherence (Lieveld et al., 2013). According to a review, there is a tendency for males with HCV to be more adherent than females (Mathes et al., 2014). Although inconclusive, this tendency is consistent with recent findings in the wider chronic illness population. In addition, women were reported as being less likely to receive long-term medical treatment and to engage in clinically recommended monitoring (Manteuffel et al., 2014). These factors are equally relevant in the new treatment landscape because they are independent of treatment duration.

Furthermore, HCV-related morbidity and mortality impose a great burden on societies and public health services. These insights may allow for the design of targeted interventions being aware of personal variables linked to the HCV patient profile before treatment, helping to maximize intervention efficacy and behavior modification, in order to prevent non-adherence and treatment failure. Additionally, psychological and neurocognitive factors are also important for more complex combinations of HCV enzyme inhibitors, becoming increasingly important for achieving treatment success as resistant mutations may develop (Gritsenko and Hughes, 2015).

Research suggests that depression in HCV-infected patients may be under-diagnosed, thereby increasing the risk of treatment discontinuation and depression severity, being that HCV itself is a risk factor for depression (Fialho et al., 2017).

The research presented herein has several limitations. The convenience sampling method and the relatively small sample sizes, particularly for women, is a major limitation. Time-since-diagnosis in a wide range and the non-existence of a matched control group are also relevant limitations. However, to compensate for this limitation, we used the values adjusted for the Portuguese population (education and age-adjusted) for the tests applied. Also, since the majority of patients were naïve at the time of sample collection, we did not control for previous treatments. These results will require verification in larger samples, and our conclusions must be interpreted with caution and are not generalizable to all HCV-infected patients.

Despite these limitations, this study has important strengths. The “hepatitis C brain syndrome” is a very interesting issue and there is a lack of studies exploring potential gender differences regarding this syndrome, using a representative sample. In contrast to the many studies that have relied solely on self-reported symptoms scales, this study used both when assessing the severity of depressive symptoms. The clinician administrated instruments were applied by a single trained clinical psychologist, preventing bias on assessment. In addition, this study also used culturally adapted and validated measures for assessing psychological and neuropsychological symptoms, and all patients were followed in a single center. Lastly, the results of this study might also have important implications for clinical practice. These findings are also important to encourage mental health professionals to take an active role in HCV treatment in the post-interferon era (Chasser et al., 2017).

## Conclusion

In conclusion, chronic HCV infection is known to be associated with neuropsychiatric dysfunction. Recognition of neurocognitive symptoms is important before and during the treatment of these patients, but it is noteworthy that neurocognitive and neuropsychiatric symptoms (depression, anxiety, and cognitive disorders) may be associated with direct HCV neurotoxicity.

According to our investigation, gender seems to have an influence on depression, anxiety and some of the indicators of cognitive functions, in chronic HCV-infected patients before treatment.

## Data Availability Statement

The datasets generated for this study are available on request to the corresponding author.

## Ethics Statement

The studies involving human participants were reviewed and approved by Centro Hospitalar Universitário Lisboa Norte ethics committee (Ref. 0536). The patients/participants provided their written informed consent to participate in this study.

## Author Contributions

All authors conceived and designed the work, revised the manuscript critically for important intellectual content, and approved the final version of the manuscript to be submitted. DB, RF, and SO conceived of the study and participated in the design and coordination. RM, MB, and SO reviewed this manuscript. IF analyzed the data.

## Conflict of Interest

The authors declare that the research was conducted in the absence of any commercial or financial relationships that could be construed as a potential conflict of interest.
